# Eptinezumab for the preventive treatment of episodic and chronic migraine: a narrative review

**DOI:** 10.3389/fneur.2024.1355877

**Published:** 2024-03-08

**Authors:** Pablo Irimia, Sonia Santos-Lasaosa, Patricia Pozo-Rosich, Rogelio Leira, Julio Pascual, José Miguel Láinez

**Affiliations:** ^1^Clínica Universidad de Navarra, Pamplona, Spain; ^2^Aragon Institute for Health Research (IIS Aragon), Hospital Clínico Universitario Lozano Blesa, University of Zaragoza, Zaragoza, Spain; ^3^Headache Unit, Neurology Department, Vall d'Hebron University Hospital, Barcelona, Spain; ^4^Headache and Neurological Pain Research Group, VHIR, Universitat Autònoma Barcelona, Barcelona, Spain; ^5^Department of Neurology, Headache Unit, Hospital Clínico Universitario, Clinical Neurosciences Research Laboratory, Health Research Institute of Santiago de Compostela (IDIS), University of Santiago de Compostela, Santiago de Compostela, Spain; ^6^Hospital Universitario Marqués de Valdecilla, Universidad de Cantabria and IDIVAL, Santander, Spain; ^7^Department of Neurology, Hospital Clínico Universitario, Universidad Católica de Valencia, Valencia, Spain

**Keywords:** eptinezumab, clinical development, narrative review, chronic migraine, episodic migraine, preventive treatment, calcitonin gene-related peptide, fast onset of action

## Abstract

Eptinezumab, a monoclonal antibody that targets calcitonin gene-related peptide (CGRP), was recently approved in Europe for the prophylactic treatment of migraine in adults who have at least four migraine days a month. Eptinezumab is administered by intravenous infusion every 12 weeks. During recent months, a considerable amount of evidence from eptinezumab trials has been published. The aim of this review is to describe the existing evidence on the tolerability, safety and efficacy of eptinezumab in patients with migraine. Data from randomized (PROMISE-1, PROMISE-2, RELIEF and DELIVER) and open-label (PREVAIL) phase 3 clinical trials have demonstrated the favorable effect of eptinezumab in migraine symptoms from first day of treatment. These studies showed that eptinezumab results in an overall reduction in mean monthly migraine days (MMDs), increases in the ≥50% and ≥ 75% migraine responder rates (MRRs) and improvements in patient-reported outcome measures in both patients with episodic migraine (EM) and with chronic migraine (CM), including patients who failed previous preventive treatments. The RELIEF trial also showed that eptinezumab, within 2 h of administration, reduced headache pain, migraine-associated symptoms and acute medication use when administered during a migraine attack. Eptinezumab benefits manifested as early as day 1 after dosing and with the subsequent doses lasted up to at least 2 years. Treatment-emergent adverse events reported by ≥2% of patients included upper respiratory tract infection and fatigue. Current evidence demonstrates that eptinezumab has a potent, fast-acting, sustained migraine preventive effect in patients with EM and CM. Eptinezumab has also shown to be well tolerated, supporting its use in the treatment of patients with migraine and inclusion in the current migraine therapeutic options.

## 1 Introduction

Migraine is a chronic neurological disorder that affects approximately 15% of the world’s population, with certain European countries presenting the highest prevalence rates (1, 2). Patients with migraine suffer recurring attacks of headache and other symptoms (including nausea, vomiting, phonophobia and photophobia) lasting approximately 4 to 72 h (3). Migraine impairs the physical, psychological and social well-being of patients, both during attacks and on migraine-free days (‘interictal’), and substantially reduces their ability to work or go to school and their health-related quality of life (HRQoL) (4–6). The burden of migraine is not only placed on patients and their families but also on society at large.

The International Classification of Headache Disorders 3 (ICHD-3) criteria defines chronic migraine (CM) as ≥15 days with migraine per month [d/mo] where at least 8 of those have migraine features for at least 3 months (7, 8). Both episodic migraine (EM) and CM can be treated using acute and/or preventive therapies. Drugs targeting the calcitonin gene-related peptide (CGRP), a peptide involved in migraine pain generation that is released by the trigemino-vascular system during migraine attacks, have emerged during the last decade. These anti-CGRP agents have demonstrated benefits for the preventive treatment of EM and CM (9–12) and a more favorable benefit–risk ratio than established treatments (13). Eptinezumab is a potent monoclonal antibody targeting the circulating CGRP, administered by intravenous (IV) infusion (14). Eptinezumab has shown to be effective and safe during the clinical development program, leading to the approval, in February 2020 in the United States and in January 2022 in Europe, of the 100 mg and 300 mg doses for the prophylactic treatment of migraine in adults with at least 4 migraine days per month (15, 16).

During recent months, a large number of results from post-hoc analyses of data from eptinezumab pivotal trials (17, 18) has been published (19–30), together with primary and post-hoc results from the DELIVER trial (31–34). Also, numerous clinical trials on eptinezumab are currently being conducted (35–42). This article provides an updated narrative review of eptinezumab evidence in EM and CM and a summary of the ongoing trials. To this end, a systematic search for articles, abstracts, and poster presentations, published prior to 12^th^ May 2023 was conducted on PubMed (U.S. National Library of Medicine), using the terms ‘eptinezumab’ and ‘ALD403’. We filtered by article type (clinical trials, randomized clinical trials and meta-analysis were included; books and documents, reviews and systematic reviews were excluded), and a total of 48 articles emerged (35 publications of results from clinical trials, including post-hoc analysis, and 13 meta-analysis). Ongoing studies were searched on clinicaltrials.gov (U.S. National Library of Medicine) using the term ‘eptinezumab’; the search resulted in 109 ongoing studies. Further publications were revised and included to provide context.

In this narrative review, we provide results of the efficacy and safety of the doses of eptinezumab that have been tested, with a special focus on the 100-mg dose, since it is the dose recommended by the health authorities. Results for the primary endpoint and key secondary endpoints of each study are presented. This review aims to build upon existing reviews of eptinezumab (43–45) by providing updated information from recently published studies and offering a detailed description of ongoing research.

## 2 Pharmacology

Eptinezumab is the only antibody targeting the CGRP pathway with an intravenous route of administration. Pharmacokinetic analyses (46) showed that the maximum concentration (C_max_) was immediate (i.e., 30 min after the start of a 30-min administration), which might provide an explanation for the fast onset of eptinezumab effect (46, 47), and contrasts with longer median values (4–14 days) of subcutaneous (SC) anti-CGRP (48–50). The eptinezumab elimination half-life of 27 days and the low exposure metrics required to achieve 90% of the maximal efficacy (EC_90_) supports its administration every 12 weeks. Its clearance (0.15 L/day) and volume of distribution (3.64 L) were consistent with other monoclonal antibodies. Age, sex, race, immunogenicity and concomitant preventive treatments only caused small changes in pharmacokinetics, and dose adjustments are not required.

In terms of route of administration, IV infusions have shown higher C_max_ than SC and intramuscular administrations (47). The rapid attainment of high plasma concentrations with the IV administration together with the good tolerability (even when co-administered with SC sumatriptan) and low immunogenicity, supported the administration of eptinezumab as IV infusions (47).

The immediate, effective and sustained effect of eptinezumab can be explained by its structure and binding properties. The attributes of the fragment antigen-binding (Fab) region and the conformational changes that occur upon eptinezumab binding to CGRP create a ‘latch-and lock’ mechanism. This mechanism hampers its dissociation (51), increases the specificity and durability of its effect, and probably underlie the sustained migraine preventive effect.

## 3 Efficacy

**Figure 1:**
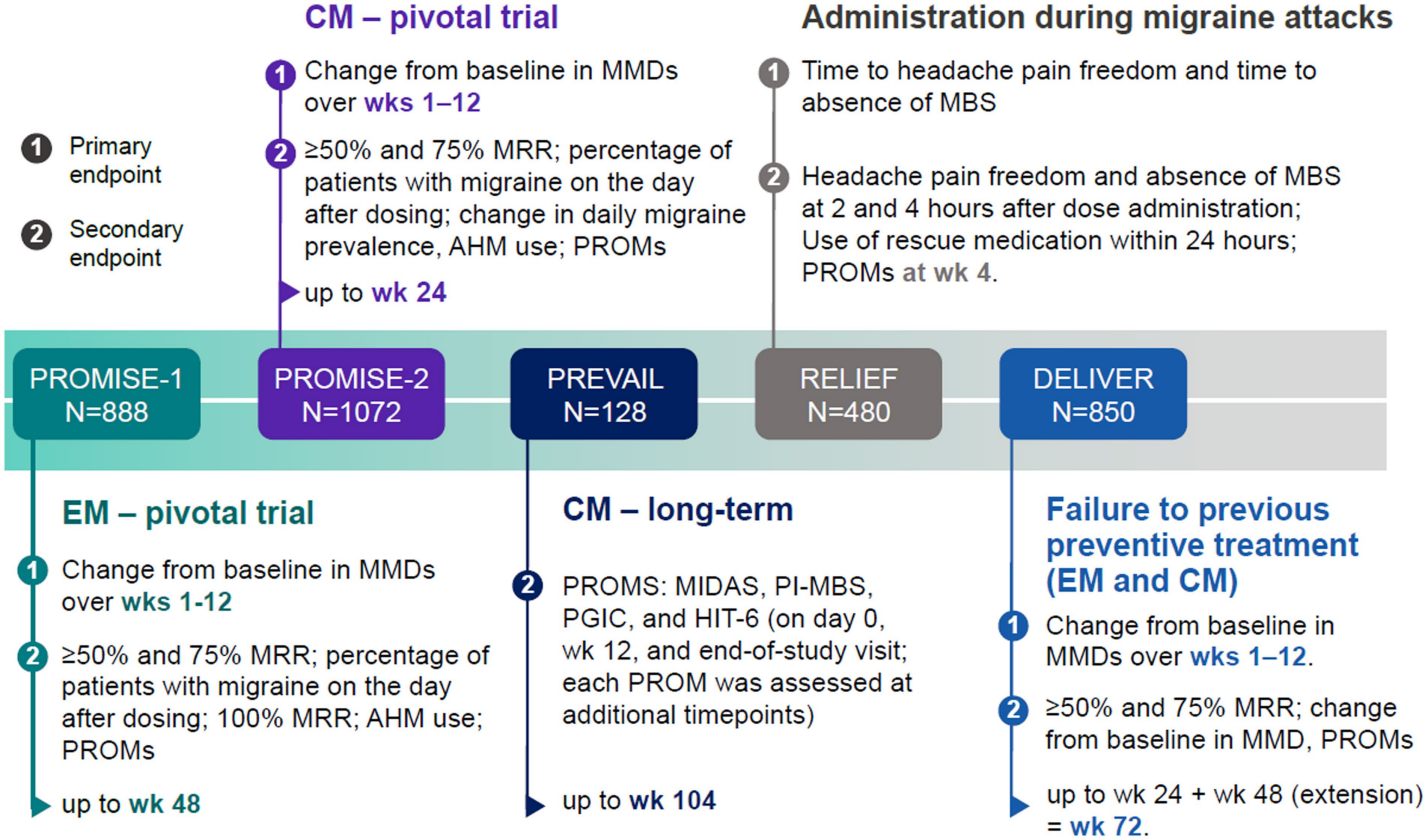
Primary and key secondary efficacy endpoints in eptinezumab phase 3 clinical trials. Primary (1) and main secondary (2) efficacy endpoints in eptinezumab clinical trials included (i) change from baseline in monthly migraine days (MMDs), (ii) ≥50% or ≥ 75% migraine responder rate (MRR), defined as the proportion of patients who achieved a ≥ 50% or ≥ 75% reduction in average MMDs at a specific week from their baseline, (iii) percentage of patients with a migraine on the day after dosing, and (iv) patient-reported outcome measures (PROMs) on disability, function, and health-related quality of life (HRQoL), among others. The number of migraine days was recorded using an electronic diary (eDiary). In the RELIEF trial, patients experiencing migraine on 4–15 days per month in the 3 months prior to screening were included. AHM, acute headache medication; CM, chronic migraine; EM, episodic migraine; MBS, most bothersome symptoms.

The efficacy and safety of eptinezumab in the prevention of migraine was evaluated in five large-scale phase 3 clinical trials: four randomized clinical trials (PROMISE-1, PROMISE-2, RELIEF, and DELIVER) and one open-label trial (PREVAIL). In this section, efficacy findings from these clinical trials and subsequent *post-hoc* analysis from these studies have been summarized. We report findings of studies including patients with EM only, followed by studies including patients only with CM, and then including both. Figure 1 displays the primary and key secondary efficacy endpoints of the phase 3 trials. Tolerability and safety data are reported in the next section of this article (see Tolerability and safety).

### 3.1 Episodic migraine

PROMISE-1 was a phase 3, multicenter, randomized, double-blind, placebo-controlled, parallel-group trial (18). Adults (*n* = 888) with EM were randomly assigned in a 1:1:1:1 ratio to receive eptinezumab 30, 100, 300 mg, or placebo for up to 48 weeks (four doses in total). Acute headache medication (AHM) was limited to ≤14 d/mo, and preventive treatment was not allowed, except for menstrual migraines. Mean monthly migraine days (MMDs) were significantly reduced from baseline (≈8.6) over weeks 1–12 compared to placebo in the 100 mg (−3.9; *p* = 0.0182) and 300 mg groups (−4.3; *p* = 0.0001). Efficacy was maintained up to week 48 (year 1), with a significant reduction in the MMDs after the second (weeks 13–24), third (weeks 25–36), and fourth (weeks 37–48) infusions (52). Overall, the percentage of patients with a ≥ 50% or ≥ 75% reduction in MMDs up to week 48 was higher with eptinezumab than with placebo. Eptinezumab-treated patients were more likely to achieve ≥50% and ≥ 75% migraine response during weeks 1–48 than placebo, except for the ≥75% migraine responder rate (MRR) which was similar (≈39%) for the 100 mg and placebo groups for weeks 37–48. Results of MMDs and MRRs from the PROMISE studies for the 100 mg dose are shown in Table 1.

**Table 1:** Summary of efficacy results of eptinezumab 100 mg from the phase III PROMISE-1 and -2 trials.

	Episodic migraine		Chronic migraine	
	PROMISE-1		PROMISE-2	
	(12 weeks)	(13–24 weeks)	(12 weeks)	(13–24 weeks)
	Ashina et al. 2020	Smith et al. 2020	Lipton et al. 2020	Silberstein et al. 2020
Change in MMD vs. baseline				
Mean	−3.9	−4.5	−7.7	−8.2
Difference from placebo (95% CI)	−0.69 (−1.25, −0.12)	−0.76 (−1.40, −0.11)	−2.0 (−2.9, −1.2)	− 1.98 (− 2.94, − 1.01)
*p* value vs. placebo	0.0182	NA	<0.0001	< 0.001
≥50% MRR				
N, (%)	110 (49.8)	137 (62.0)	205 (57.6)	217 (61.0)
Difference from placebo (95% CI)	12.4 (3.2, 21.5)	10.6 (1.5, 19.8)	18.2 (11.1, 25.4)	17.0 (9.8, 24.1)
P value vs. placebo	0.0085^a^	NA	<0.0001	< 0.001
≥75% MRR				
N, (%)	49 (22.2)	74 (33.5)	95 (26.7)	140 (39.3)
Difference from placebo (95% CI)	6.0 (−1.4, 13.3)	8.7 (0.3, −17.1)	11.7 (5.8, 17.5)	15.6 (8.9, 22.2)
P value vs. placebo	0.1126	NA	0.0001	≤ 0.001

Results by 12-week and 24-week dosing interval are showed. ^a^Not statistically significant per the testing hierarchy; unadjusted value of p presented. CI, confidence interval; MMD, monthly migraine days; MRR, migraine response rate; NA, not available.

The migraine preventive effect of eptinezumab in patients with EM was observed on the first day after the infusion (18, 53). The average percentage of patients with a migraine on any given day at baseline (30.7%) was significantly reduced on day 1 with the 100-mg dose (14.8%; *p* = 0.0312) and the 300-mg dose (13.9%, *p* = 0.0159) compared with placebo (22.5%), entailing a > 50% reduction compared to baseline. The mean number of MMDs on day 1 was −4.5 with 100 mg (*p* = 0.021) and − 4.7 (*p* = 0.010) with the 300 mg (53). However, it should be acknowledged that the specific assumptions made by the researchers for evaluating this endpoint represent a limitation. The study normalized measures to a 28-day month to facilitate comparison with MMDs, based on the assumption of a consistent migraine rate over this period, which may not be entirely acceptable in clinical practice.

Eptinezumab also reduced AHM use from weeks 1–4 up to week 24, especially in patients with higher baseline use, and improved HRQoL in three domains of the Short-Form Health Survey (SF-36; bodily pain, physical role-functioning, and social role-functioning) after 1 year (52).

### 3.2 Chronic migraine

PROMISE-2 was a phase 3, multicenter, randomized, double-blind, placebo-controlled, parallel-group trial (17). Adults with CM (*n* = 1,072) were randomly assigned in a 1:1:1 ratio to receive eptinezumab 100, 300 mg, or placebo every 12 weeks for up to 24 weeks (two infusions in total). Use of barbiturates or prescription opioids were allowed ≤4 d/mo if use was stable for ≥2 months. Other AHM such as triptans, nonsteroidal anti-inflammatory drugs, and simple analgesics were not restricted. The reduction in MMDs from weeks 1–12 was statistically significant for eptinezumab doses of 100 (−7.7; *p* < 0.0001) and 300 mg (−8.2; p < 0.0001) compared to placebo (−5.6). Patients receiving eptinezumab were more likely than the placebo group to achieve ≥50% and ≥ 75% migraine response during weeks 1–12 (see Table 1).

As observed in patients with EM (18), eptinezumab also had a migraine-preventive effect on day one after dosing in patients with CM (17). The average percentage of patients with a migraine on any given day at baseline (58%) was significantly reduced on the first day after administering 100 mg (−50.3%; *p* < 0.0001) and 300 mg (−51.6%; *p* < 0.0001) of eptinezumab, but not placebo (−21.7%). Patients using eptinezumab vs. placebo reported greater improvements in the ability to function (measured by the 6-item Headache Impact Test; HIT-6) and higher reductions in AHM use from baseline to week 12 (17).

Results through 24 weeks of treatment showed that the reductions in MMDs observed with the first dose (17) continued to improve after the second dose (−8.2 days with 100 mg and − 8.8 with 300 mg, vs. −6.2 with placebo, *p* < 0.0001) (54). The ≥50% and ≥ 75% MRRs also increased after the second dose (54). The percentage of patients with no migraine for a 4-week interval (from week 13 to 24) was higher in the eptinezumab groups (100 mg: 17.8%; 300 mg: 20.8%) than in the placebo group (9.3%). Both doses further reduced AHM days from baseline to up to week 24 (difference from placebo: − 1.1 days with 100 mg; −1.7 days with 300 mg).

Health authorities have suggested including the assessment of most bothersome symptoms (MBS) as an endpoint in clinical trials evaluating migraine treatment (55). In line with this recommendation and considering that MBS vary from person to person, individual MBS were secondarily assessed in the PROMISE-2 study (56). Patients were asked to self-report their MBS in an open-ended question (patient-identified MBS, PI-MBS) at screening and to rate the overall change in their severity during subsequent visits. At week 4, a higher percentage of eptinezumab-treated patients reported *much* or *very much improvement* in their PI-MBS (100 mg, 45%; 300 mg, 57%) compared to placebo (29%). Improvements in PI-MBS continued at week 12 (100 mg, 53%; 300 mg, 61%; placebo, 34%), and were sustained up to week 32. A *post-hoc* analysis showed that improvements in PI-MBS correlated with changes in MMDs and other patient-reported outcome measures (PROMs), such as HIT-6, Patient Global Impression of Change [PGIC], SF-36, and EuroQol-5-dimension visual analog scale (EQ-5D-5L VAS) at week 12 (20). PI-MBS improvement predicted better outcomes across all PROMs in addition to change in the MMDs (*p* < 0.003) (20). PI-MBS, thus, appear to be a clinically useful patient-centered measure for evaluating the effect of preventive treatments in those aspects more disturbing to each individual.

Several *post-hoc* analyses of data from clinical trials further exhibited eptinezumab efficacy in alleviating CM (Table 2). Buse et al. (19) assessed the consistency and predictive ability of migraine response during month 1 on later response, by using data from the PROMISE-2 study. During month 1, more eptinezumab-treated than placebo-treated patients were ≥ 75% MRR (100 mg, 30.9%; 300 mg, 36.9%; placebo, 15.6%) and had improvements in the PGIC scores (100 mg, 46%; 300 mg, 58.7%; placebo 32.4%). Among ≥75% migraine responders at month 1, the ≥75% MRR for the five subsequent months was higher in the eptinezumab groups (100 mg, 41.8%; 300 mg, 46.5%) than in the placebo group (36.8%). PGIC scores at month 1 predicted scores at month 6, with more than 80% of the patients who obtained *very much improved* rates at month 1 obtaining *very much improved* or *much improved* scores at month 6 (100 mg, 82.9%, 300 mg, 86.4%; placebo, 81.5%). Overall, patients who benefited from eptinezumab the first month had consistent benefits during the following months, suggesting that early response can predict sustained response.

**Table 2:** *Post-hoc* analysis of clinical trials in CM.

Objective	Main findings	Reference
Data from the PROMISE-2 study		
To assess the effect of eptinezumab on PROMs in patients with CM and MOH	Eptinezumab resulted in clinically meaningful improvements in mean HIT-6 total scores by week 4 and were sustained up to week 24. Eptinezumab was also associated with numerically greater improvements in the PI-MBS and SF-36 scores compared with placebo.	Starling et al. (57)
To examine the relation between changes in PI-MBS and changes in MMDs and other PROMs	Improvements in the PI-MBS correlated with changes in MMDs and in the PROMs (PGIC, HIT-6, EQ-5D-5L VAS, and SF-36) at week 12. The magnitude of treatment effects on PI-MBS was greater for eptinezumab groups than for placebo.	Lipton et al. (20)
To evaluate the general consistency and predictability of early treatment response on later response	The percentage of patients who achieve ≥75% MRR at Month 1 and up to Month 6 was higher in eptinezumab-treated patients than in placebo-treated patients. Most patients attaining *very much improved* scores (PGIC) at Month 1 also had *much* or *very much improved* scores for all 5 subsequent months.	Buse et al. (19)
To evaluate the relationship between headache frequency reductions and changes in AHM use	Eptinezumab-treated patients presented fewer days with both headache and AHM use than placebo-treated patients. Triptans were the type of AHM that presented the highest reduction of use.	Cowan et al. (21)
To examine changes in the occurrence, severity, and symptoms of headache episodes	Monthly severity and frequency of headache days and episodes decreased more in eptinezumab-treated groups than in the placebo group throughout 24 weeks.	McAllister et al. (22)
To assess change in MMDs from baseline through 24 weeks in patients with a dual diagnosis of CM and MOH	A higher decrease in MMD occurred in patients treated with eptinezumab compared to placebo from week 1–24. Around half of patients receiving eptinezumab (*vs* one third receiving placebo) were below CM and MOH thresholds throughout this period.	Dienner et al. (58)
Data from the PREVAIL study		
To assess item-level changes in the MIDAS over 2 years	Eptinezumab reduced headache days, headache pain severity, absenteeism and presenteeism at week 12 up to week 104.	Blumenfeld et al. (23)

AHM, Acute headache medication; EQ-5D-5L VAS, EuroQol-5-dimension visual analog scale; HIT-6, 6-item Headache Impact Test; MIDAS, Migraine Disability Assessment Test; MMD, monthly migraine days; MOH, medication-overuse headache; PGIC, Patient Global Impression of Change; PRO, patient-reported outcomes; SF-36, Short-Form Health Survey.

Forty percent (*n* = 431) of patients in the PROMISE-2 study had medication-overuse headache (MOH), a secondary headache resulting from the overuse of analgesics or other medication to alleviate acute migraine attacks. Subgroup analyses in these patients with dual diagnosis of CM and MOH were conducted (57–59). Greater mean changes from baseline in MMDs were observed for eptinezumab 300 mg during week 1 through 12, compared with placebo (*p* < 0.0001) and the changes were sustained up to week 24 (58). Approximately half and one-third of eptinezumab-treated patients experienced ≥50% and ≥ 75% migraine response, respectively, as early as weeks 1 and up to week 24, compared with one-third and one-sixth of placebo patients, respectively. Fewer patients had a migraine on day 1 in eptinezumab groups (27.8%, 100 mg; 30.1%, 300 mg), than in the placebo group (45.5%). Slightly more than half of patients (51.1%, 100 mg; 54.4%, 300 mg) receiving eptinezumab were below the diagnostic thresholds for CM for 24 weeks, compared with one third (32.4%) of patients receiving placebo. Similar percentages were observed on the use of AHM below the diagnostic thresholds for MOH (50.5%, 100 mg; 49.5%, 300 mg; 27.1%, placebo), suggesting resolution of both diagnoses in half of the treated patients. Over 24 weeks, monthly AHM use almost halved. Eptinezumab also numerically increased the percentage of patients who were below ICHD-3 diagnostic thresholds for CM and MOH (59). These results suggest that patients with CM and MOH could have their MOH managed with eptinezumab, without requiring withdrawal from pharmacological treatment.

The association between reductions in headache frequency and changes in AHM use was specifically tested in another subanalysis (21). Three subgroups of CM patients were considered: total population, patients with MOH, and patients with MOH who were ≥ 50% responders during treatment (weeks 1–24). In the three subgroups, the proportion of days with both headache and AHM use decreased more in eptinezumab-treated patients compared to placebo-treated patients (total population: 25.1% vs. 17.0%; MOH subgroup: 29.2% vs. 18.4%; MOH with ≥50% response subgroup: 38.3% vs. 31.5%). Triptans were the type of AHM that presented the highest reduction of use during treatment.

McAllister et al. showed that eptinezumab not only steadily decreased migraine and headache frequency, but also severity, with the remaining headache episodes being less severe (*post-hoc* analysis of the PROMISE-2) (22). This reduction in headache frequency and severity was maintained up to week 104 by post-hoc analysis of another phase 3 trial (see *Safety* section for a full description of the PREVAIL study); these analysis also revealed that absenteeism and presenteeism decreased during this two-year period as well (23).

### 3.3 Episodic and chronic migraine

**Figure 2:**
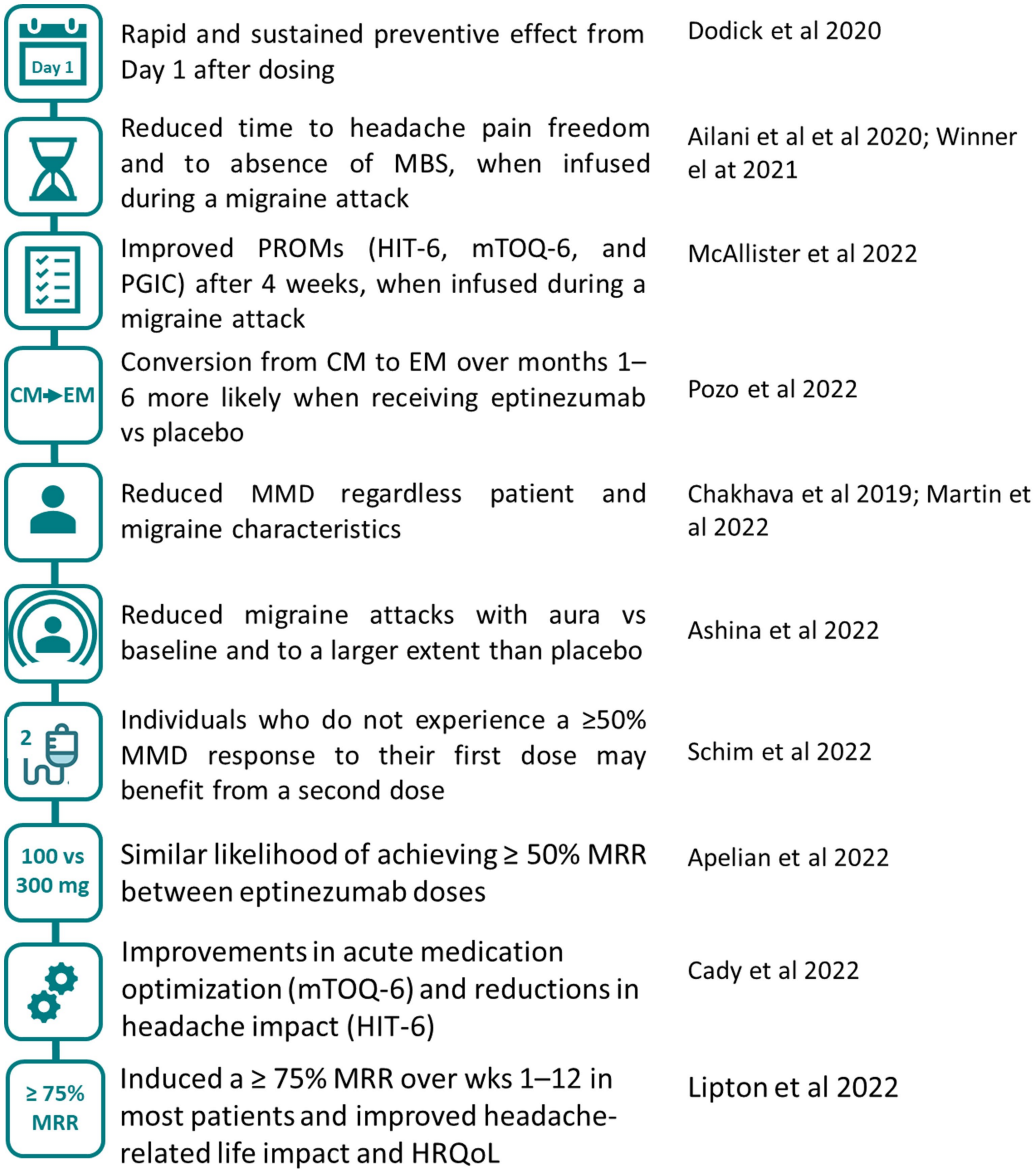
Effect of eptinezumab on migraine (EM and CM). Main conclusions regarding eptinezumab efficacy from *post-hoc* analysis that pooled patients from the pivotal trials (26–30, 53, 60, 61) and from studies that included patients with migraine 4–15 d/mo (24, 25, 62, 63). HIT-6, 6-item Headache Impact Test; HRQoL, health-related quality of life; MMD, monthly migraine days; MRR, migraine response rate; mTOQ-6, 6-item migraine Treatment Optimization Questionnaire; PGIC, Patient Global Impression of Change; PROMs, patient-reported outcome measures.

The efficacy of eptinezumab treatment separately observed in either EM or CM was further confirmed with a broader spectrum of migraine patients (Figure 2). Here, we report the main results of *post-hoc* analyses of pooled data of the PROMISE-1 and -2 studies and findings from the RELIEF and DELIVER trials.

The efficacy of eptinezumab on the first day following infusion observed on EM (18, 64) or CM (17, 65) separately was further confirmed when data from these trials was pooled and analyzed together (53). Moreover, the value attributed by patients to the reduction in the likelihood of migraine on day 1 after infusion was tested by an online survey in patients with EM or CM (*n* = 101). Patients considered the time to effect onset to be at least as important as a clinically relevant reduction in migraine days during the first month of treatment (66). Another online survey (*n* = 604) also showed that patients deemed speed of onset, together with mode of administration and durability of preventive effect, as the treatment attributes with the highest relative importance (67). Early onset and sustained efficacy of eptinezumab could potentially increase treatment adherence, since one of the main reasons for treatment discontinuation is perceived lack of efficacy (68).

Considering the rapid effect of eptinezumab and its relevance to patients, the efficacy of eptinezumab was evaluated in patients who were having a migraine attack in the RELIEF study, a phase 3, multicenter, parallel-group, double-blind, randomized, placebo-controlled trial (62, 63). The study included 480 patients eligible for preventive migraine treatment (experiencing migraine 4–15 d/mo) who were treated either with 100 mg of eptinezumab or placebo within 1 to 6 h of the migraine attack onset. Participants were not allowed to use rescue medication 24 h prior to or 2 h following the infusion. Eptinezumab obtained better results for time to headache pain freedom (median hours, 4 eptinezumab vs. 9 placebo; *p* < 0.001) and time to absence of MBS (2 eptinezumab vs. 3 placebo; *p* < 0.001) (63). As early as 1 h after the start of the infusion, more eptinezumab-treated patients than placebo-treated achieved headache pain freedom, headache pain relief, and absence of MBS (*p* < 0.05), which lasted through hour 48 (62). After 2 h, almost one-fourth of patients (23.5%) treated with eptinezumab achieved headache pain freedom (62). Median time to next migraine was also significantly longer with eptinezumab (10 days) than with placebo (5 days; *p* < 0.001) (63). Fewer patients used AHM within 24 and 48 h in the eptinezumab than in the placebo group (*p* < 0.001) (63). Patients eligible for preventive migraine treatment, therefore, could benefit from receiving eptinezumab during an active migraine attack, due to the rapid resolution of migraine symptoms and the reduction of AHM use.

Eptinezumab treatment initiated during a migraine attack also resulted in greater improvements on PROM scores compared to placebo, such as in HIT-6 (*p* < 0.0001), 6-item migraine Treatment Optimization Questionnaire (mTOQ-6; *p* < 0.01), and PGIC (*much* or *very much improved*: 59.3% eptinezumab vs. 25.9% placebo) after 4 weeks (24). The improvements in PROM scores were more pronounced in patients with headache pain freedom 2 h after the infusion (24) and in those with poor acute treatment optimization prior to eptinezumab (25).These findings suggest that at least some of the benefits reported by patients after 4 weeks of the initial dose might be related to the fast onset of eptinezumab effect.

Diagnostic migraine classification, based on the number of headache days, continues to play a major role in determining access to preventive treatment. Pozo-Rosich et al. conducted a *post-hoc* analysis of data from the PROMISE studies considering four classification categories of monthly headache days (MHDs: ≥ 15, CM; 10–14, high-frequency EM; 4–9, low frequency EM; and ≤ 3) (26). The analysis showed that eptinezumab reduced headache frequency over months 1–6. Patients treated with eptinezumab improved their diagnostic classification, as they were more likely than placebo-treated patients to experience a shift of one or more headache day categories, achieving a reduction of at least one MHD over 6 months. More than half of CM patients were classified as EM the first month after eptinezumab infusion (58.7%, 100 mg; 61.7%, 300 mg), whereas less than half of placebo patients (45.6%) were. Similarly, a higher percentage of patients in the eptinezumab than in the placebo group converted from CM to EM in the following 6 months and were also more likely to fall below the threshold for initiating preventive treatment (≤ 4 MHDs).

Importantly, eptinezumab efficacy was independent of patient and disease characteristics at baseline, as demonstrated by several *post-hoc* analyses of the PROMISE studies. Efficacy was observed regardless of the race/ethnicity, demographics, migraine features, and concomitant medication use (28, 60, 69, 70). The efficacy of eptinezumab demonstrated no dose-dependent effect within the range of 100 to 300 mg. Apelian et al. (27) reported that the likelihood of achieving ≥50% MRR was similar across patients with either the 100-mg or 300-mg eptinezumab dose. The odds ratio, however, indicated that 300 mg had slightly higher efficacy than 100 mg in certain subgroups (such as patients with EM and obesity; and patients with CM and: < 15 MMDs at baseline, mobility problems on the EQ-5D-5L in CM, or a score > 45 on the SF-36 vitality), but these numerically higher improvements for the 300-mg dose were not statistically significant. These results were aligned with a meta-analysis of four randomized controlled trials (*n* = 2,739) that found no statistically significant differences between the two doses in ≥50% MRR, MMDs, and patients with migraine 1 day after the infusion.

Around half of the patients in the PROMISE trials had a self-reported history of experiencing aura. Ashina et al. (29) assessed the efficacy of eptinezumab in this subgroup. This analysis confirmed that eptinezumab decreased MMDs in patients with aura and EM (−4.0, 100 mg; −4.2, 300 mg; −3.1–7.1) or CM (−7.1, 100 mg; −7.6, 300 mg; 6.0, placebo), similar to results in the global study population. Eptinezumab reduced, to a greater extent than placebo, the percentage of migraine attacks with aura relative to baseline, suggesting a potential effect on lessening aura frequency beyond migraine frequency. The reduction of migraine attacks with aura may be important because migraine with aura is associated with an increased risk of cerebrovascular and cardiovascular disease (71, 72).

In the pivotal trials, the effect of eptinezumab in patients with failure with previous preventive treatment remained unexplored, despite patients being refractory to several preventive treatments are common. To evaluate eptinezumab in patients with failure with prior treatments, Ashina et al. conducted the DELIVER study, a phase 3b, multicenter, randomized, double-blind, placebo-controlled trial (31). Patients with EM (54%; high-frequency EM, 41%) or CM (46%) and two-to-four previous preventive treatment failures were included and were randomly assigned to eptinezumab 100, 300 mg, or placebo (1:1:1). The most common previous preventive medication failures were amitriptyline and topiramate. The reduction in mean MMDs from baseline to weeks 1–12 was −4·8 with 100 mg, −5·3 with 300 mg, and − 2·1 with placebo (*p* < 0·0001), and the effect was sustained or further improved over weeks 13–24 (−5.4 with 100 mg, −6.1 with 300 mg, and − 2.4 with placebo). The ≥50% MRR was also greater (*p* < 0.0001) for eptinezumab (42% with 100 mg; 49% with 300 mg) than placebo (13%) over weeks 1–12 and the effect further improved after the second infusion (52% with 100 mg; 59% with 300 mg; and 24% with placebo). The ≥75% MRR followed a similar trend over this period (weeks 1–12: 16% with 100 mg, 19% with 300 mg, 2% with placebo; weeks 13–24: 21% with 100 mg, 28% with 300 mg, 7% with placebo; *p* < 0.0001). Even in these patients with failure with prior treatments, total scores in the HIT-6 had higher improvements with eptinezumab compared with placebo by week 12, and fewer severe migraine attacks, MHDs and episodes, and monthly acute medication days (12–4 weeks).

Analysis of subpopulations considering sex, age, disease classification, MOH diagnosis, or number of prior preventive treatment failures, confirmed a significantly greater efficacy of eptinezumab than placebo for reducing MMDs across most subgroups (32, 33), demonstrating its efficacy regardless patient’s characteristics. Moreover, eptinezumab significantly improved more than placebo patients’ overall health, headache-related quality of life, absenteeism, presenteeism, work productivity loss, and activity impairment (32, 34).

Results from the DELIVER study consolidated the fast and sustained efficacy of eptinezumab observed in prior studies. However, findings from the DELIVER study are worth discussing here. On one hand, the placebo effect in the DELIVER study was numerically lower than in the PROMISE studies, which might reflect lower expectations among patients with failure of previous preventive treatments compared with those without prior failure. The efficacy observed with eptinezumab, despite the low expectations for these patients, highlights its superiority vs. placebo. On the other hand, slightly lower ≥50% and ≥ 75% MRR were registered in the DELIVER than in the PROMISE studies, probably due to a more difficult-to-treat migraine in patients included in former than in the latter. Yet, a higher proportion of patients with unsuccessful prior preventive therapies and treated with eptinezumab had higher ≥50% MRR than when treated with other SC anti-CGRP (73). The DELIVER study had a dose-blinded extension (48 weeks) which demonstrated long-term effectiveness and safety and tolerability of eptinezumab for up to 18 month in patients with migraine and 2–4 prior preventive treatment failures (74).

One of the most recent *post-hoc* analyses of the PROMISE studies by Schim et al. showed that around one-third of patients with suboptimal response (<50% MMD reduction from baseline in MMDs over weeks 1–12) after the first dose, responded (≥ 50% MMD reduction) to the second dose (30). They identified potential first-infusion predictors of second-infusion response by using full logistic regression analysis. The analysis showed that percent change in MMDs across weeks 1–12 was a significant first-dose predictor of second-dose response in patients with EM and CM, and change in HIT-6 total score in CM. Even in patients who had no change in MMDs after their first infusion, the likelihood of having a ≥ 50% MMD increased after the second infusion. Schim et al. also included a table with the likelihood of response according to first-dose predictors (30), which could be helpful for making individualized treatment decisions in the clinical practice.

Several aspects of the efficacy results of the studies reported here deserve to be discussed. First, some of these findings have been obtained from *post-hoc* analysis, which entail methodological limitations, and should be replicated in studies specifically designed to test those endpoints. Second, a considerable placebo effect was observed across the efficacy endpoints, in line with findings of other anti-CGRP (75–77) and migraine treatments (78). As it is well known, the placebo effect could be due to several factors, including the clinical environment, interactions with healthcare professionals, patient expectations and beliefs, or the treatment administration route (79). Third, all data was obtained from clinical trials, which included strict screening criteria for patients and were conducted in a highly controlled clinical environment. Studies in the real-world setting are needed to confirm results from clinical trials in a more heterogeneous population in terms of demographics, socioeconomic factors, migraine characteristics, comorbidities, and concomitant medication use. Also, the efficacy of eptinezumab in patients with previous treatment failure to an anti-CGRP has not been examined since these patients were excluded from the DELIVER study. The efficacy of eptinezumab in this population, as well as in combination with other prevention and nonpharmacological therapies, should be explored.

## 4 Tolerability and safety

The tolerability and safety of eptinezumab was also evaluated in the above-mentioned clinical trials (17, 18, 23, 31, 52, 54, 62–65, 80), which showed that eptinezumab was well tolerated and had a favorable safety profile in patients with EM and CM. Treatment-emergent adverse events (TEAE) were similar across doses and treatment groups and did not increase with continuous dosing. These results were further confirmed in two meta-analyses (81, 82). One of these meta-analyses included patients who received at least 1 dose of eptinezumab (*n* = 2076) or placebo (*n* = 791) in five eptinezumab trials (NCT01772524, NCT02275117, PROMISE-1, PROMISE-2, and PREVAIL). The study showed that the proportion of patients who had at least one TEAE was consistent across active and placebo groups (54.0%, 30 mg; 52.2%, 100 mg; 56.7%, 300 mg; and 52.3%, placebo) and no dose-related trends were observed (Table 3) (81). The most frequent study-drug-related TEAE was fatigue (eptinezumab, 2.0% vs. placebo, 0.9%) (81). Among study-drug-related TEAEs, six events reported by four patients (eptinezumab, *n* = 3; placebo, *n* = 1) were considered severe. No life-threatening (grade 4) or fatal (grade 5) TEAEs occurred (81). The other meta-analysis that pooled 2,739 participants from four of the eptinezumab trials (NCT01772524, NCT02275117, PROMISE-1, PROMISE-2) confirmed that TEAEs were not statistically different between the eptinezumab and placebo group (82).

**Table 3:** Summary of TEAEs.

Patients, n (%)	Pooled analysis of 5 clinical trials (TEAEs with incidence of ≥2% of patients in any subgroup)			Deliver trial (TEAEs with incidence of ≥1.5% of patients in any subgroup)		
	Eptinezumab		Placebo (*n* = 791)	Eptinezumab		Placebo (*n* = 298)
	100 mg (*n* = 701)	300 mg (*n* = 823)		100 mg (*n* = 299)	300 mg (*n* = 294)	
Any TEAE	366 (52.2)	467 (56.7)	414 (52.3)	127 (42)	120 (41)	119 (40)
Any study-drug–related TEAE	92 (13.1)	124 (15.1)	74 (9.4)	NA	NA	NA
Any serious TEAE	11 (1.6)	17 (2.1)	11 (1.4)	5 (2)	7 (2)	4 (1)
Any TEAE leading to infusion interruption	11 (1.6)	19 (2.3)	6 (0.8)	1 (<1)	3 (1)	0
Any TEAE leading to discontinuation	9 (1.3)	19 (2.3)	8 (1.0)	1 (<1)	6 (2)	1 (<1)
TEAEs with incidence of ≥1%						
Upper respiratory tract infection	45 (6.4)	64 (7.8)	48 (6.1)	–	–	–
COVID-19	–	–	–	20 (7)	17 (6)	16 (5)
Nasopharyngitis	44 (6.3)	72 (8.7)	41 (5.2)	5 (2)	9 (3)	3 (1)
Dizziness	27 (3.9)	16 (1.9)	21 (2.7)	2 (1)	4 (1)	5 (2)
Fatigue	20 (2.9)	24 (2.9)	13 (1.6)	2 (1)	6 (2)	4 (1)
Diarrhea	NA	NA	6 (0.8)	0	5 (2)	5 (2)
Nausea	NA	NA	26 (3.3)	4 (1%)	5 (2%)	4 (1%)
Urinary tract infection	–	–	–	1 (<1)	5 (2)	4 (1)
Upper abdominal pain	–	–	–	5 (2)	4 (1)	2 (1)
Arthralgia	–	–	–	5 (2)	4 (1)	0
Back pain	–	–	–	5 (2)	3 (1)	4 (1)

Pooled analysis of the NCT01772524, NCT02275117, PROMISE-1, PROMISE- 2, and PREVAIL (primary treatment phase only) studies. Clinical trials included percentage with one decimal place (81), whereas DELIVER trial rounded up percentages and did not report any decimal places (31). In the pooled analysis of five clinical trials, diarrhea and nausea were reported in 30 (1.4%) and 69 (3.3%) patients, respectively, in all eptinezumab doses (1,000 mg, 300 mg, 100 mg, 30 mg, and 10 mg), but data for the 100 mg and 300 mg doses, separately, was not available (81). The ‘–‘symbol indicates that no mention on that event was made in the study article.

Up to 1 year, a lower proportion of patients reported at least one TEAE in the second and subsequent dosing intervals than in the first dosing interval across all treatment groups, and no serious tolerability issues were identified with continued dosing (52). The PREVAIL study, an open-label phase 3 trial, assessed long-term safety and immunogenicity of eptinezumab 300 mg, a dose that is three times higher than the recommended dose according to the Summary of Product Characteristics (SmPC), for up to eight doses in patients with CM and a follow-up of 104 weeks (80). During the study, 91 out of 128 patients experienced at least one TEAE, most of them during the first treatment phase. Thirteen patients had severe TEAEs, which were unrelated to eptinezumab. Eighteen patients had at least one study-drug-related TEAE, with hypersensitivity and fatigue being the most common. Only one serious TEAE was considered related to eptinezumab (anaphylactic reaction, grade 2; however, due to the patient’s medical history and reaction during the TEAE, the investigators concluded that this event could be more accurately described as an allergic reaction).

Importantly, eptinezumab has been shown to be safe and well tolerated regardless of patient characteristics (28, 31, 83). Two randomized, double-blind, placebo-controlled trials were conducted to examine the safety and metabolic effects of eptinezumab in patients without migraine who were overweight or obese (study 1; *n* = 24) and with type 1 diabetes (T1D; study 2; *n* = 21) (83). In the first study, 37.5% of eptinezumab-treated and 25.0% of placebo-treated patients reported a TEAE; only one TEAE (in the active group) was deemed drug-related. No difference between patients treated with eptinezumab and placebo emerged from baseline to day 7 in terms of basal metabolic rate. In the second study, 85.7% of eptinezumab-treated and 100% of placebo-treated patients reported a TEAE, which were drug-related in four patients (two in the active group, two in the placebo group). No significant difference in insulin sensitivity was detected between treatment groups. In both studies, all TEAEs were mild or moderate in severity and no TEAEs leading to discontinuation or serious events were reported.

Results from the DELIVER study also confirmed that TEAEs occurred with a similar incidence and seriousness in patients treated either with eptinezumab or placebo who had two-to-four previous preventive treatment failures (31). The most common TEAE was COVID-19 in all groups (Table 3).

Patients with migraine have an increased risk for cardiovascular events, which might be potentially exacerbated by anti-CGRP treatments that hamper vasodilation (84). The effect of eptinezumab on cardiovascular events and comorbidities was assessed by pooling safety data (*n* = 2,268) from four clinical trials on eptinezumab (85). The analysis showed that cardiovascular TEAEs considered treatment-related were rare (*n* = 27) and the incidence was similar in placebo (*n* = 6) and eptinezumab groups (100 mg, *n* = 8; 300 mg, *n* = 7; 1,000 mg, *n* = 6). Most cardiovascular TEAEs occurred in patients with ≥1 and ≥ 2 cardiovascular risk factors at baseline, and all events were mild or moderate in severity with none being serious or life-threatening. No relevant effect of eptinezumab or difference between treatment groups was observed on blood pressure, heart rate or concomitant cardiovascular medication use for up to 56 weeks. A total of 9 (1.3%) and 13 (1.9%) patients receiving the 100-mg and 300-mg dose and 8 (1.0%) patients in the placebo group discontinued study drug due to TEAEs.

The immunogenic profile of eptinezumab was also characterized by pooling data (*n* = 2076) from five clinical trials (NCT01772524, NCT02275117, PROMISE-1 and -2, and PREVAIL studies) (86). Immunogenicity was assessed in serum from all patients who received eptinezumab, and samples were collected on day 0 and regularly throughout each study at similar time points (2-week time point to evaluate early seroconversion, followed by sampling at 4-week intervals). Anti-drug antibody (ADAs) and neutralizing antibodies (Nabs) were found in 15.9 and 6.2% patients treated with eptinezumab, respectively. In all four trials, ADA/NAb development began around week 8, peaked at week 24 (second infusion), and steadily declined during the subsequent weeks, regardless of eptinezumab dose or number of doses. Even when ADA or NAb presented, eptinezumab maintained its efficacy and safety profile.

Collectively, results from pooled data from clinical trials has demonstrated that eptinezumab, in general, is well tolerated, and has a favorable long-term safety profile.

## 5 Potential advantages of eptinezumab and ongoing studies

Eptinezumab has unique features that offers potential clinical benefits. It shows a fast onset of action and effectiveness in treating migraine attacks. Using eptinezumab during a migraine attack leads to significant improvements in pain freedom and the most bothersome symptoms within the first 2 h post-infusion (63). Moreover, the convenience of intravenous administration every 3 months distinguishes eptinezumab from other monthly subcutaneous CGRP mAbs. Existing literature suggests that individuals in need of rapid relief, those who prefer less frequent dosing, or those who have not responded adequately to other CGRP mAbs may benefit the most from eptinezumab. However, comprehensive comparative studies are necessary to establish specific patient profiles.

Nine clinical trials on eptinezumab are currently ongoing (35–42, 87). A brief description of each study is reported in Table 4. Most of the studies are randomized clinical trials in adults, with change in the number of MMDs at variable timepoints as the primary endpoint. The CHRONICLE trial is an open-label study evaluating primarily the safety of eptinezumab in patients with cluster headache.

**Table 4:** Ongoing studies on eptinezumab registered on clinical trials.

Study design	Status	Planned N	Diagnosis / Age	Primary endpoint	Identifier / Name
Observational, prospective (3 years)	Recruiting	500	HFEM and CM (aged 18–75)	Change in the number of MMDs from baseline to month 3–6–9-12	NCT05570149 EMBRACE (87)
Interventional, randomized, double-blind, parallel-group, placebo-controlled	Recruiting	570	Migraine and MOH (aged 18–75)	Change in the number of MMDs from baseline to weeks 1–4	NCT05452239 RESOLUTION (35)
Interventional, randomized, double-blind, parallel-group, placebo-controlled delayed-start	Completed	304	eCH (aged 18–75)	Change from baseline in the number of weekly attacks, averaged over weeks 1–2	NCT04688775 ALLEVIATE (36)
Interventional, randomized, double-blind, parallel-group, placebo-controlled	Recruiting	513	CM (aged 18–75)	Change in the number of MMDs from baseline to weeks 1–12	NCT04921384 SUNRISE (37)
Interventional, randomized, double-blind, parallel-group, placebo-controlled	Recruiting	285	CM (aged 12–17)	Change from baseline in number of MMDs averaged over weeks 1–12	NCT04965675 PROSPECT-2 (38)
Long-term, open-label (dose-blinded), extension of the PROSPECT-2 and 19357A study	Recruiting	610	EM or CM (aged 6–17)	Number of patients with TEAEs from baseline up to week 44	NCT05164172 REJOIN (39)
Interventional, open-label, fixed-dose, long-term extension of the SUNRISE	Enrolling	100	CM (aged 18–75)	Number of participants with AEs from baseline up to week 68	NCT05064371 (40)
Interventional, open-label, fixed-dose multiple administration	Active, not recruiting	131	cCH (aged 18–75)	Number of participants with AEs from baseline up to week 56	NCT05064397 CHRONICLE (41)
Open-label, single-dose, pharmacokinetic	Active, not recruiting	28	EM or CM (aged 6–17)	AUC (0-infinity) eptinezumab from dosing to week 20	NCT04537429 (42)

AEs, adverse events; AUC, Area under curve; cCH, chronic cluster headache; eCH, episodic Cluster Headache; EM, episodic migraine; MMDs, monthly migraine days; MOH, medication-overuse headache; The ‘-‘symbol indicates that no mention on the name was made on the website.

Clinical trials on children and adolescents are also ongoing and will provide information on the pharmacokinetics, efficacy, and safety of eptinezumab on these populations, who are currently not allowed to be treated with eptinezumab according to the SmPC.

In terms of eptinezumab dosage optimization, further research is needed to discern specific patient profiles that would benefit from the 300 mg dose, particularly in cases where the recommended 100 mg dose results in a suboptimal response. Such studies are key for assisting neurologists in making informed dosing adjustments, particularly in light of the current absence of detailed guidance in the SmPC.

## 6 Conclusion

Collectively, data from clinical trials has demonstrated that eptinezumab is a potent, fast, effective and safe preventive treatment across migraine diagnoses, patient demographics and disease characteristics, and prior treatments. Eptinezumab benefits manifested as early as day 1 after dosing and lasted up to at least 2 years. The evidence reported by the studies supports that eptinezumab is effective as a preventive treatment and also demonstrates immediate efficacy in alleviating migraine pain and symptoms when administered during a migraine attack in candidates eligible for preventive treatment. Therefore, the data suggest that eptinezumab could serve as a quarterly preventive treatment option, offering potential additional benefits for active migraine attack when initiated during the attacks. The rapid onset and sustained efficacy of eptinezumab could potentially reduce migraine burden and give patients a sense of control. Real-world studies that assess the effectiveness and safety of eptinezumab in a diverse patient population, and in comparison with other CGRP mAbs, are needed. This research would increase our understanding of the role of eptinezumab in migraine management.

## Author contributions

PI: Conceptualization, Funding acquisition, Validation, Writing –review & editing. SS-L: Validation, Writing –review & editing. PP-R: Validation, Writing –review & editing. RL: Validation, Writing –review & editing. JP: Validation, Writing –review & editing. JL: Conceptualization, Funding acquisition, Validation, Writing –review & editing.

## Glossary

AEs: adverse events
AHM: acute headache medication
cCH: chronic cluster headache
CI: confidence interval
CGRP: calcitonin gene-related peptide
CM: chronic migraine
C_max_: maximum concentration
d/mo: days per month
eCH: episodic cluster headache
eDiary: electronic diary
EM: episodic migraine
EC_90_: 90% of the maximal efficacy
HIT-6: 6-item Headache Impact Test
HRQoL: health-related quality of life
ICHD: International Classification of Headache Disorders
IV: intravenous
MBS: most bothersome symptoms
MHD: monthly headache days
MIDAS: Migraine Disability Assessment Test
Min: minutes
MMDs: monthly migraine days
MOH: medication-overuse headache
MRR: migraine responder rate
mTOQ-6: 6-item migraine Treatment Optimization Questionnaire
PGIC: Patient Global Impression of Change
PI-BMS: patient-identified BMS
PROMs: patient-reported outcome measures
SC: subcutaneous
SF-36: Short-Form Health Survey
SmPC: Summary of Product Characteristics
TEAE: treatment-emergent adverse event
