# Supplementary figures and images for: Eptinezumab for the preventive treatment of episodic and chronic migraine: a narrative review
Source: Front Neurol. 2024 Mar 8;15:1355877. doi: 10.3389/fneur.2024.1355877 (PMC10959239; doi:10.3389/fneur.2024.1355877)

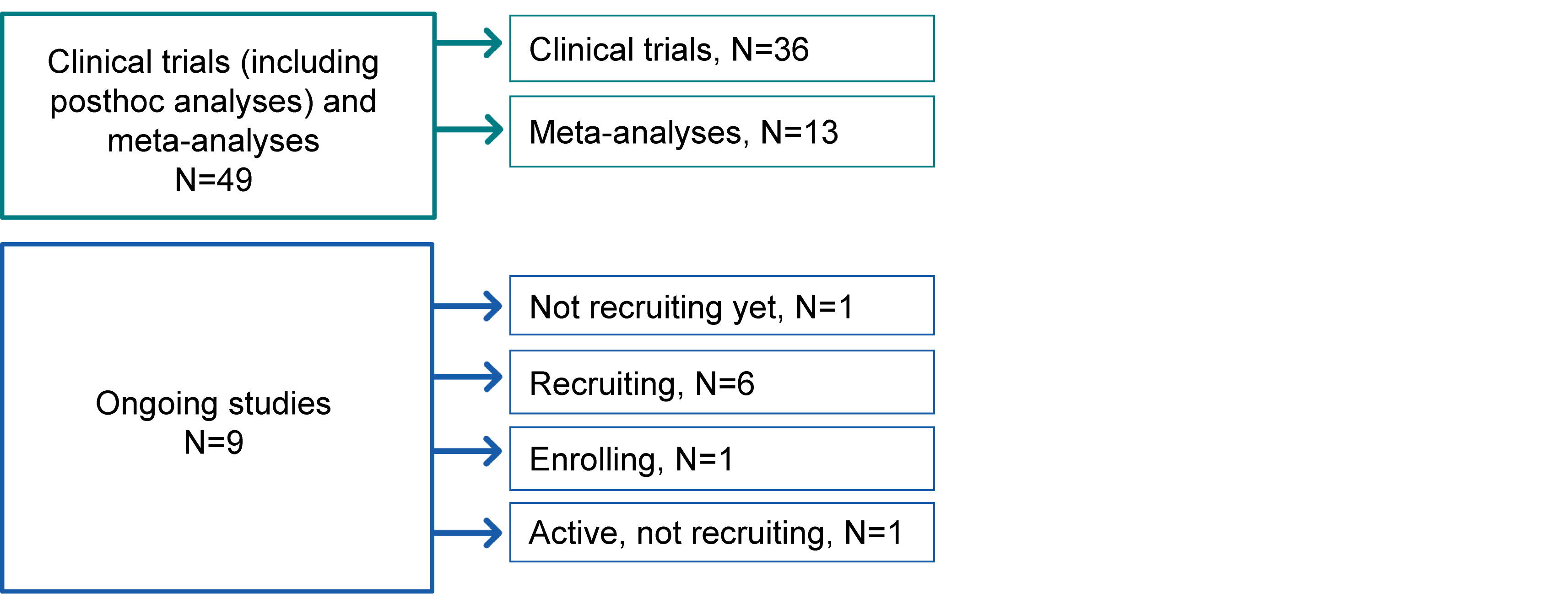

Supplement: Supplementary file 1 [file Image_1.TIF]
